# Perspectives across Canada about implementing a palliative approach in long-term care during COVID-19

**DOI:** 10.1186/s12904-023-01142-3

**Published:** 2023-03-30

**Authors:** Julia Kruizinga, Stephanie Lucchese, Shirin Vellani, Vanessa Maradiaga Rivas, Sandy Shamon, Karine Diedrich, Laurel Gillespie, Sharon Kaasalainen

**Affiliations:** 1 School of Nursing, McMaster University, Hamilton, ON Canada; 2 Department of Family Medicine, Division of Palliative Care, McMaster University, Hamilton, ON Canada; 3 Canadian Hospice Palliative Care Association, Ottawa, ON Canada

**Keywords:** Long term care, Palliative approach to care, COVID-19

## Abstract

**Background:**

Long-term care (LTC) homes have been disproportionately impacted during COVID-19.

**Purpose:**

To explore the perspectives of stakeholders across Canada around implementing a palliative approach in LTC home during COVID-19.

**Methods:**

Qualitative, descriptive design using one-to-one or paired semi-structured interviews.

**Results:**

Four themes were identified: (1) the influence of the pandemic on implementing a palliative approach, (2) families are an essential part of implementing a palliative approach, (3) prioritizing advance care planning (ACP) and goals of care (GoC) discussions in anticipation of the overload of deaths and (4) COVID-19 highlighting the need for a palliative approach as well as several subthemes.

**Conclusion:**

The COVID-19 pandemic influenced the implementation of a palliative approach to care, where many LTC homes faced an overwhelming number of deaths and restricted the presence of family members. A more concentrated focus on home-wide ACP and GoC conversations and the need for a palliative approach to care in LTC were identified.

**Supplementary Information:**

The online version contains supplementary material available at 10.1186/s12904-023-01142-3.

## Introduction

Long-term care (LTC) homes have been disproportionately impacted during COVID-19, with homes facing high proportions of deaths [1] and residents forced into prolonged social isolation [2]. These challenges have positioned an important emphasis on a palliative approach to care, which aims to improve the quality of living and dying for those with life-limiting illnesses through psychological, social, spiritual, and physical support [3]. Few LTC homes have established and embedded palliative approaches to care [4] and implementing these approaches is difficult as cited in numerous articles, even prior to COVID-19 [5–7].

A palliative approach to care is a way of caring for patients with life-limiting illnesses that integrates the principles of palliative care into care at all stages of an illness and across sectors and disciplines, not just at end of life (EOL) [8]. A palliative approach “does not link the provision of care too closely with prognosis” and includes early conversations about values and wishes, well before EOL to help prepare people better for EOL decisions [9]. More pronounced during the COVID-19 pandemic were reportedly fewer visits from healthcare professionals (i.e., physicians), less contact with family members and friends as a result of visitor restrictions, and fewer hospital transfers due to system burdens [10]. With the rise in COVID-19 cases in LTC homes and associated deaths alongside disruption to the sector in providing a palliative approach to care [1, 11], it is more critical than ever to address strengthening a palliative approach in LTC homes. Given the unprecedented challenges faced by LTC homes during COVID-19, it is imperative to learn from stakeholders’ experiences on implementing a palliative approach during the COVID-19 pandemic. It will be an important first step in uplifting LTC homes’ capacity to integrate a palliative approach with chronic disease management as a standard of care and to better prepare for future outbreaks and disasters.

Given the immediate need for LTC homes to build capacity to implement a palliative approach during COVID-19, a joint initiative between Strengthening a Palliative Approach in Long-Term Care and the Canadian Hospice Palliative Care Association unfolded to gather information, resources, and tools across Canada from key experts in the field. The goal was to compile a toolkit for LTC homes to quickly access and meet their needs in implementing a palliative approach to care. In March and April 2021, the Strengthening a Palliative Approach in Long-Term Care research team conducted an environmental scan. The scan examined current Canadian LTC practice as it pertains to a palliative approach to care, including advance care planning (APC) and goals of care (GoC) discussions, in the environment of COVID-19. Therefore, the purpose of this study was to explore the perspectives of stakeholders across Canada around implementing a palliative approach in LTC home during COVID-19.

## Methods

### Design

This study used a qualitative descriptive approach to explore stakeholders’ perspectives [12, 13]. Qualitative descriptive studies draws from naturalistic inquiry where the researcher explores a phenomenon in its natural state with the aim to discover how meaning is interpreted by individuals, how individuals construct meaning within their social world and what meaning they attribute to their experiences [12]. This approach was appropriate for the purpose of conveying meaning and comprehension by describing the perspectives of stakeholders on implementing a palliative approach in LTC during a pandemic [14]. Ethics approval was obtained from Hamilton Integrated Research Ethics Board (HiREB), #13,140.

### Sampling and recruitment

Stakeholders were purposively recruited with the goal to include representation from all provinces and territories in Canada. Both purposeful and snowball sampling were used and the estimated sample size was based on the aim to recruit 30 stakeholders for the environmental scan [15]. Representatives from local, provincial, and federal organizations were sought to participate if they could provide knowledge about palliative approach standards and practices in LTC. These stakeholders needed to be English or French-speaking individuals who had experience working in or with LTC homes during the COVID-19 pandemic and be aged 16 years or older. Thirty-seven stakeholder interviews were held between March 2021 and April 2021.

### Data collection

For data collection, stakeholders completed a short demographic questionnaire (i.e., age, gender, their profession/professional title, length of time in role). Stakeholders also took part in a one-to-one or paired semi-structured interview (Supplementary file 1) with a research staff member using Zoom Video Conferencing. Zoom is a HIPAA compliant web and video conferencing platform and was selected due to its ease of use, cost-effectiveness, and security and privacy features [16]. The duration of the interviews lasted between 30 and 60 min. Stakeholders were encouraged to email or verbally share any resources during their interview related to the environmental scan and those were collected and stored in a repository.

### Data analysis

All interviews were audio recorded, and transcribed verbatim before being entered into Dedoose V9.0.46 to aid in data management and analysis. Qualitative data were compiled and coded using content analysis, which aims to draw meaning from the collected data and elicit realistic conclusions [17]. The first three transcripts were independently coded by two researchers (JK, SL) and after coding the initial transcripts, the authors (JK,SL) met with the larger team to discuss, validate and address conflicts of the data (JK, SL, SV, VMR, SK). Once intercoder agreement and interpretations of the data was confirmed, the lead author (JK) completed analysis of the remaining transcripts, while meeting with the co-authors to ensure agreement. Similar codes were grouped into major topic categories and summarized to form main themes.

## Results

### Characteristics of the Sample

Of the 37 individuals who participated in an interview, 29 stakeholders completed the demographics form. Representation from all provinces and territories was achieved with the exception of Nunavut, despite multiple attempts. The majority of stakeholders identified as female (86%), with ages ranging from 27 to 68 years. The average number of years worked in LTC was 11 years and years worked ranged from one to 27 years. Stakeholders reported on their primary profession or job which was grouped into the following categories: advanced practice nurse (n = 7), long-term care management (n = 9), physician (n = 6), professor (n = 7), registered healthcare professionals (n = 8) and organizational lead (n = 3). For employment conditions, stakeholders reported they were funded by either government (n = 16), other (n = 14), or a mixed combination of government, grant-funded and/or other (n = 3). The majority of stakeholders were working full-time during COVID-19 (90%).

### Overview of findings

Four main themes were summarized from stakeholder interviews (Table 1). Many stakeholders noted that there were pre-existing efforts in integrating a palliative approach to care in their LTC home, but regardless, the *influence of the pandemic on implementing a palliative approach to care (1)* was widespread with navigating the ever-changing guidelines, dealing with staffing shortages and restricting interdisciplinary members from entering homes. *Families were identified as essential to implementing a palliative approach (2)* and their absence was felt during the pandemic. Practices were adapted to include families and stakeholders had the opportunity to reflect on the role of families in LTC homes. In the environment of COVID-19, many LTC homes anticipated the pronounced effects of the virus to result in an increased number of deaths which led to *the prioritizing ACP and GoC discussions* (3). Dedicated staff were charged with this task and supports were developed to aid in implementing a palliative approach to care. Lastly, stakeholders reflected on the fact that *COVID-19 highlighted the need for a palliative approach in LTC (4)*. There were identified gaps as LTC homes were faced with mass causalities within this increasingly frail and vulnerable population. A palliative approach was defined as continuous work and the next steps for their LTC homes were defined as stakeholders had reflected on all that occurred during COVID-19 and how a palliative approach to care was implemented.

**Table 1:** Themes and subthemes

Theme	Subtheme	Additional Quotes
1. Influence of the pandemic on implementing a palliative approach	Pre-existing efforts to integrate a palliative approach to care	*“Well we had finished out two year palliative approach project and we had finished the implementation plan just as COVID came. So all of our care homes had training. The vast majority of our physicians had training. So, it was implemented in all the care homes as much as they were letting it seep into their daily practices” (P8, BC)*
	Keeping up with the guidelines	*“But, it’s been that constant trying to keep up with the latest, you know not only the Ministry and Public Health guidelines but also the…the guidelines that are coming down from your own facility and it’s been caused in flux and a lot of anxiety I think, just worrying about, are we going to get it? Can we manage it? You know those are the things” (P5, ON)*
	COVID-19 and chronic staffing challenges	*“I mean we just got absolutely overwhelmed. I mean it was just too many people getting sick, so few staff to look after them” (P7, ON)*
	Restrictions to entering homes limiting interdisciplinary care	*“I mean we do actually have a pain and symptom management nurse that before COVID was coming into the homes I think once a month to kind of review patients and give some ideas and that was done in all facilities in our areas. With COVID that kind of stopped because you don’t really want people wandering around. So that’s been kind of unfortunate because I think that at least that was something that was somewhat helpful” (P5, ON)*
2. Families are an essential part of implementing a palliative approach	Family absence impacted the delivery of a palliative approach to care	*“with visitor restrictions and families not being as freely able to come into the sites that really limited our times or those opportune moments to have important conversations that weren’t so formal” (P9, BC)*
	Adapting practice to involve families	*“so when we admit somebody we admit their family and they become part of the whole care program. So, for us when we were able to open up and have families come back. We called them our essential care partners. […] So, they come in person and go through a two hour training session before they come in to visit. That has been a great tool throughout this so that they can come in and feel comfortable. For staff to see that they’re doing that they feel comfortable letting the doors back open again right” (P1, NB)*
	Opportunity to reflect on the role of family in long-term care	*“I think some of what I see for the most pressing need for long term care is the fact that it’s a family thing. This is about not just an individual and the tasks they need to kind of keep going from day to day it really is about a person embedded in their family and their community. So, long term care is about you know the resident, their connections and their intertwinement with their family and their community and that really palliative care should…palliative care and long term care, the long term care needs to see that as a whole” (P1, SK)*
3. Prioritizing ACP and GoC discussions in anticipation of the overload of deaths	Increased frequency of ACP and GoC discussions	*“It accelerated it for everybody. I would assume that the great majority of patients now have had goals of care conversations that are timely and appropriate for the situation just because COVID necessitated that. I think, and again as I said before something very…decision maker or patient understands the reality of that goals of care discussion. Something real in their face that they can understand and experience” (P8, BC)*
	Dedicated staff for home-wide ACP and updating GoC	*“We had RNs that contacted every resident, every family member and then had discussions about you know is this the care that you want? Is there things you want changed knowing that COVID could be here? Is there different things that you would like to have? And that gave us the opportunity to tell them the things that we could do in long term care if they chose to stay there” (P1, PEI)*
	Developing supports for a palliative approach to care	*“I mean from my perspective and just because I was involved with a lot of the provincial planning and that with the end of life order set, I really felt that that got a huge push to have something like that was very strongly…you know encouraged that that be created very quickly because that guidance was needed at those times” (P3, SK)*
4. COVID-19 highlighted the need for a palliative approach	Identified gaps in a palliative approach to care	*“we don’t have a strong emphasis on advanced care planning. Obviously, there’s the establishment of the goals of care but that seems to be as far as most homes in our region have gone. So definitely some more support, and some tools and some education I think would be very beneficial and hopefully we’ll get there” (P1, NS)*
	Influence of COVID-19 on building momentum around a palliative approach	*“So, until we make that…it truly more of a place where you can live and die in quality. Even if we have a good conversation, if we don’t have the capacity to enact that care then we’re not improving anything. That has to do with staffing levels and training and capacity and the way long term care is respected and a whole bunch of bigger things” (P1, QB)*
	Defining a palliative approach to care as continuous work	*“so the pandemic has highlighted the need for palliative care but it’s also taken us back in terms of people understanding what palliative care is” (P6, ON)*

### Influence of the pandemic on implementing a palliative approach

#### Pre-existing efforts to integrate a palliative approach to care

Many stakeholders noted that there were pre-existing efforts to integrate a palliative approach to care in their LTC homes. This factor helped to support the needed efforts of a palliative approach during COVID-19 where many residents were dying, and deaths far exceeded what LTC homes were used to. One stakeholder mentioned:

“We have been working with integrated palliative care and palliative care within the province so we have a provincial strategy. We were working on it long before COVID….about two years ago. So, that was really a benefit that we had that. We had been working on an order set for palliative care and we had introduced that order set to one of our homes as a pilot and they started using it. But when we started hearing about COVID, palliative care order set was actually introduced in all of our homes” (P1, PEI).

#### Keeping up with the guidelines

While many LTC homes were prepared with pre-existing efforts to establish palliative approach programs, LTC homes faced challenges one way or another. The ongoing policy and directives changes from the Public Health Agency of Canada and provincial guidelines and at an organizational level were one major influence during COVID-19. One stakeholder reflected on the ever-changing guidelines and their impact on staff trying to implement a palliative approach:

“The rules were evolving quickly and so much change for them. Just even things that you know, they felt so much pressure and so much stress around their responsibilities to protect residents” (P1, NL).

The need to protect both residents and staff brought about tension and stress and an additional layer of work with implementing a palliative approach to care.

#### COVID-19 impacts on chronic staffing challenges

The pandemic-related environment also enhanced the chronic staffing challenges faced by LTC homes. One stakeholder commented on the second-tiered nature a palliative approach became:

“And a palliative approach is considered an extra. But when you’re short staffed and everyone is you know isolated and sick and you’re worried and stressed then it’s not an immediate need. Which I gather around Christmas time that’s what it was like here, they were pretty stretched” (P1, AB).

Another stakeholder commented on the way staff felt stretched and tried to do their best despite working in dire circumstances:

“Let’s just say…even there where everybody is aware that dying is happening, staff were not always feeling like they could be by the bedside for a person. In the normal circumstances. Now you’ve got five people dying on your floor and they’re dying by themselves, their family is not around. It has to have completely exacerbated that sense of distress for the staff. That they couldn’t be there for those final moments and they couldn’t offer that sort of check in every hour kind of care that they know not everybody wants but many people want. That they can’t even do in regular life, where they tried…you know what staff do is they come in on their own time to do this kind of thing. You know they’re covering…you know sort of filling the gaps. The staff had more work and then people were dying and staff weren’t able to be there by their bedside” (P1, QB).

#### Restrictions to entering homes limiting interdisciplinary care

LTC home also needed to respond to who would be allowed entrance to their homes in the environment of COVID-19. As a result, many LTC homes enacted restrictions that limited interdisciplinary care by external consultants and partners and their contributions to a palliative approach to care. One stakeholder commented on the barriers to a palliative approach to care these decisions caused:

“Well and in general right, they were trying to eliminate anybody who wasn’t absolutely essential right? So, even I know even the physician groups right? They tried to limit the number of physicians going in and consultants going in. […] So, they lost some supports that they would typically be able to rely on right? Because we were minimizing the number of people that could come in” (P2, AB).

### Families are an essential part of implementing a palliative approach

#### Family absence impacted the delivery of a palliative approach to care

All stakeholders emphasized that families are an essential part of implementing a palliative approach to care. Stakeholders commented on how the absence of family impacted the delivery of a palliative approach to care:

“it was so difficult on so many layers. […] And that’s really our goal you know with palliative care, is for our clients to die as peacefully as they can and…but also to have the families at the bedside so they can experience that too. So COVID changed the colour of the water under the bridge. And not having families there certainly made it that much more difficult. So, I would say if anything that’s what we all struggled with, is that piece of not having families able to be there” (P3, NB).

Stakeholders also commented on the different psychosocial impacts on residents by limiting family members to the LTC home:

“early part of it we were like okay batten down the hatches. Nobody in or out. You know we have to ensure all this other stuff, you know protect people. But we didn’t consider what would be the social, emotional impact of doing this on residents and families” (P1, NL).

#### Adapting practice to involve families

Though family members were limited in a physical sense to being present in many homes, stakeholders noted that many LTC homes adapted their practice to involve families as part of a palliative approach to care:

“one thing that I did struggle with as far as palliative care goes, because it is a very holistic approach…was not having the families at the bedside. […] So, we touched base with families every single day sometimes twice a day. And we would also take in, an iPad. And our patients…or our patients, our clients would have a video with their family members. So, their family members could see how they were doing” (P3, NB).

When family members were able to re-enter further into COVID-19, training was provided to ensure their safety when allowing them to visit their loved ones.

#### Opportunity to reflect on the role of family in long-term care

When discussing the role of families in LTC home during COVID-19, stakeholders had the opportunity to meaningfully reflect on the valuable role of family in LTC, particularly at end of life:

“When I say system, I mean engaging families. You know the prolonged lock out of families especially the designated family caregivers speaks, to the fact that families weren’t perceived as really having a valued role before. I think that’s one thing” (P1, SK).

The important role of families in LTC home and as part of a palliative approach was reflected on by stakeholders who identified the pressing need for the presence of families in LTC home, especially in being involved in the care team and in making decisions:

“It’s hard to engage with the family members and get them involved in decisions when they’re not actually sort of part of the care team right? Because we kind of sidelined them. So that’s…you know that’s…that was the biggest impact I think COVID had right? We removed a very important group of the care team. And whether that was right or not right?” (P2, AB).

### Prioritizing the role of ACP and GoC discussions in anticipation of the overload of deaths

#### Increased frequency of ACP and GoC discussions

In anticipating the many deaths that could occur due to the predicted severity of COVID-19, stakeholders commented on the preparedness that took place to plan accordingly, particularly with regards to ACP and GoC discussions. Many stakeholders identified the increased frequency in ACP and GoC discussions that took place in the LTC home:

“In the very beginning when we wanted to make sure that they were all updated we had RNs that contacted every resident, every family member and then had discussions about you know is this the care that you want? Is there things you want changed knowing that COVID could be here? Is there different things that you would like to have? And that gave us the opportunity to tell them the things that we could do in long term care if they chose to stay there.” (P1, PEI)

As an important aspect of a palliative approach, much foreground work was done to ensure timely conversations were had and to prepare for any potential crisis.

#### Dedicated staff for home-wide ACP and updating GoC

In order to conduct these home-wide advance care plans and update goals of care, one stakeholder noted the importance of a dedicated staff that was charged with this role:

“I think it was just having that dedicated nurse that could actually be able to spend the time and talk to all those families. I think that was pretty critical to have that person designated to do that.” (P5, ON)

These efforts were deemed as a worthy endeavour and one stakeholder commented on the need to prioritize these efforts early on from staff:

“We just did it. We did that early so we weren’t in crisis. So, myself and the resident care coordinator kind of took that on and made those phone calls. It wasn’t a lot of phone calls. We are a home of sixty elders and we do a pretty good job of advanced care planning on move in. So, every move in there’s a discussion about the advanced care plan.” (P4, SK)

#### Developing supports for a palliative approach to care

In considering the increasing need for a palliative approach for care during the pandemic, stakeholders identified supports developed in order to deliver a palliative approach to care:

“I mean from my perspective and just because I was involved with a lot of the provincial planning and that with the end of life order set, I really felt that that got a huge push to have something like that was very strongly…you know encouraged that could be created very quickly because that guidance was needed at those times.” (P3, SK)

### COVID-19 highlighted the need for a palliative approach

#### Identified gaps in a palliative approach to care

There were many efforts made during the pandemic to integrate a palliative approach to care and minimize the influences of COVID-19 in LTC and the potential disruption of existing practices. In these efforts, the need for a palliative approach and identifying gaps to sustain a palliative approach in LTC were emphasized. As one stakeholder stated:

“I think the other big thing is just exposing the gaps and that we still have work to do.” (P8, BC).

Another stakeholder commented on existing gaps:

“There are pressing needs related to palliative care of which advanced care planning and goals of care discussion is one of them. And I think that there should be the resources put into place by regional and provincial governments to build up the palliative care approach in the sector.” (P6, ON)

#### Influence of COVID-19 on building momentum around a palliative approach

As stakeholders reflected on the impact of COVID-19 on implementing a palliative approach in LTC, ongoing work and next steps were identified. As one stakeholder reflected:

“I am choosing to believe that although this was a horrible year for long term care it has shone a spotlight on what those of us who have worked in long term care for a long time have known and have been advocating for and that we’re now going to see the changes that we need to get with the times. […] I hope…like I said before that if nothing else we shone a spotlight on that and that policy makers, funders, government take a closer look at long term care and you know provide support and make some changes so that we can really provide for our elders, our seniors, our vulnerable people what they deserve.” (P2, NS)

The pressing needs identified by stakeholders highlighted the impetus for change in LTC around a palliative approach and the momentum generated to implementing a palliative approach so that residents can live and die with quality care and staff have the capacity and support to provide this care.

#### Defining a palliative approach to care as continuous work

While many LTC homes did have existing practices and procedures for a palliative approach in place, stakeholders commented on the continuous nature of a palliative approach. One stakeholder described:

“so with implementing a palliative approach over the past two years, this isn’t a once and done philosophy that can be implemented. Because it can be such a culture shift. I mean it is a bit site specific to each site. But what we learned quite early on is that this is continuous ongoing work.” (P9, BC)

## Discussion

In describing their experiences of providing a palliative approach to care during COVID-19, stakeholders were able to further describe what a palliative approach to care is and what future work is needed. The COVID-19 pandemic underscored the need for a palliative approach to care and the existing gaps and work to be done. While there is still progress to be made towards normalizing a palliative approach to care in LTC, there is momentum building and learning gained around implementing a palliative approach to care from this study that can steer the way forward.

Of primary concern was the role of family in LTC homes during COVID-19. To not recognize family as essential care partners and a necessary component of delivering a palliative approach to care earlier on was one of the greatest failures. Family members provide hands-on care and social interaction, advocate for their loved one, and bring an essential presence to the home [18, 19]. As recent guidelines have reflected, family and caregivers should have an inclusive role in implementing a palliative approach as there is the potential for serious harm and negative impacts to families and residents as a result of visitor restrictions [20]. Though families were recognized as an essential part to implementing a palliative approach to care, this was not reflected in the way families were involved in LTC homes and their absence was felt. The tension existed in following public health measures and a failure to understand the role of family in implementing a palliative approach. Much has shifted in how these important care partners were involved in care for the duration of the pandemic, and there has been much learned as to the importance of integrating family in implementing a palliative approach. Moving forward in a post-COVID-19 world and considering the defined role, family and caregivers need to be considered an essential part of caring for residents and that must be reflected in inclusive visiting policies [20]. For those without family or caregivers, consideration should also be given for staff and volunteers making visits to these residents to ensure these gaps are met in providing a palliative approach to all residents and inclusive of those with formally identified family.

As learned from stakeholders, the COVID-19 pandemic provided a unique environment to speed up work around a palliative approach to care and encourage earlier conversations around ACP and GoC [21]. Where these conversations may have not been as normalized in practice before the pandemic, the reality is that the average length of stay in LTC homes is 18 months [22] and frequent and regular conversations are important to delivering a palliative approach in LTC. While COVID-19 may have been the initial motivator to ensure these conversations were had earlier rather than later, it should not end there. Structures and processes to facilitate initial and ongoing conversations around ACP and GoC should become best practice for LTC homes [23]. While COVID-19 necessitated much of the work done, this upstream work is not only relevant to the environment of COVID-19, but an important practice reflected in a palliative approach embedded into the philosophy of LTC homes.

Finally, systemic and pre-existing challenges in LTC must be addressed in order for the work of implementing a palliative approach to continue. Issues of capacity in terms of staffing, education, and the nature of working in LTC need to be addressed. While these issues existed prior to the pandemic, they were exacerbated during it. Building capacity for LTC staff by ensuring they are supported through internal and external resources and providing ongoing education are important steps that can be taken [24, 25]. For example, a recent scoping review describes four types of implementation strategies, facilitation, education/training, internal engagement, and external engagement, as necessary for building capacity with LTC homes [6]. Making LTC homes a desirable place for recruiting and retaining staff, and demonstrating meaningful work, especially as it pertains to caring for residents at the end of life, is important to addressing and securing a sustainable LTC workforce amid an aging population. Much attention has been drawn to LTC homes during COVID-19, often negatively given the dire environments they became. However, as one of the largest and primary settings to deliver a palliative approach to care, there must be invested effort and accountable leadership while there is momentum to use these circumstances to address the systemic issues and focus on the important work and care that is happening in LTC homes. As pre-existing challenges have been identified and will continue to be expected with an aging population, there is now more than even the need to expand service delivery and strengthen a palliative approach to care in LTC [26]. In acknowledging that some efforts have been made to integrate a palliative approach in different LTC homes [25], by building on these efforts and using the momentum established, LTC homes can champion palliative approaches to care. Strengthening a palliative approach is one strong solution to bettering the environment in which residents can be supported in their quality of life alongside their family and staff who have the capacity to deliver the best care.

There are several study limitations to note. As this study focused on stakeholder perspectives, the perspectives of other LTC staff, and that of residents and families was absent. Additional research to explore the impacts of COVID-19 on a palliative approach from these perspectives would be beneficial. The interviews were conducted between March 2021 and April 2021, which may have introduced some recall bias and inaccuracies; however, this timeline supported the ability to hear perspectives representative of over a year into the pandemic. Since participation in the study was voluntary, a selection bias may also have occurred. While representation from all provinces and territories was desired, no stakeholder from Nunavut was able to be recruited, despite multiple attempts.

## Conclusion

The aim of this study was to explore the perspectives of stakeholders across Canada around implementing a palliative approach in LTC home during COVID-19. While the influence of the pandemic on implementing a palliative approach to care was identified through pre-existing challenges in LTC and an overwhelming number of deaths, a more concentrated focus on home-wide ACP and GoC discussions and focus on the need for a palliative approach to care in LTC was made. Future studies should explore the experiences of LTC staff, as well as residents and families.

## Electronic supplementary material

Below is the link to the electronic supplementary material.

Supplementary Material 1

## Data Availability

The dataset analysed during the current study is available from the corresponding author on reasonable request.

## Abbreviations

ACP: Advance Care Planning
COVID: Corona virus
EOL: End of life
GoC: Goals of Care
LTC: Long-Term Care
