# Supplementary material for: Perspectives across Canada about implementing a palliative approach in long-term care during COVID-19
Source: BMC Palliat Care. 2023 Mar 30;22:32. doi: 10.1186/s12904-023-01142-3 (PMC10060130; doi:10.1186/s12904-023-01142-3)
Supplement: Supplementary file 1 — Supplementary Material 1 [file 12904_2023_1142_MOESM1_ESM.docx]

# Semi-Structured Interview Guide

*Thank you for agreeing to participate in this interview.*

*As you may already know, we are conducting interviews with people who work with long-term care staff, as well as representatives of provincial organizations that focus on end-of-life care. Our goal is to examine what is currently happening across Canada in Long Term Care as it pertains to a palliative approach to care, including advance care planning and goals of care, in the environment of COVID-19.*

*We realize that this may be a difficult or emotional topic. All of the questions in this interview are voluntary – please tell me if there is a question you would not like to answer. Please only share what you feel comfortable sharing and tell me if you prefer not to say anymore on a topic. Our intention with this interview is to better understand about implementing a palliative approach to care, including advance care planning and goals of care, in long-term care homes during COVID.*

1. Can you please tell me about the relationship of your work to long-term care? [probe: frontline worker, administrator, partner organization]
   1. What has your experience been like working in/with long-term care during COVID?

**Implementation of a Palliative Approach to Care**

1. How has COVID-19 impacted the implementation of a palliative approach to care in long-term care?

[If needed: *Definition of a Palliative Approach to Care*: A palliative approach to care includes the management of pain and other uncomfortable symptoms. However, it also pays attention to a range of other human needs, such as the need for psychological, social, emotional, spiritual, and practical support. A palliative approach to care embraces the needs of the whole family (however defined), both during a life-limiting illness and at the time of death.]

[*Probes*: Impact of: staffing levels, staff working at different homes, organizations beyond a single LTC home, linkages with hospital or community-based response teams, infection prevention and control measures, meeting the basic care needs of residents, including feeding and bathing, medical assessment and treatment of residents affected by COVID-19 or otherwise, family caregiver access]

1. During COVID, what are the most pressing needs that exist in long-term care homes related to a palliative approach to care?
2. What, if any, specific considerations should we be aware of when considering a palliative approach to care within your region?

**Implementation of Advance Care Planning**

1. How has COVID-19 impacted the implementation of advance care planning in long-term care?

[If needed: *Definition of Advanced Care Planning*: is a way to help residents think about, talk about and document wishes for health care; is a process of reflection and communication. It is a time for to reflect on values and wishes, and to let people know what kind of health and personal care individuals would want in the future if they were unable to speak for yourself.]

[*Probes*: Impact of: staffing levels, staff working at different homes, organizations beyond a single LTC home, linkages with hospital or community-based response teams, infection prevention and control measures, meeting the basic care needs of residents, including feeding and bathing, medical assessment and treatment of residents affected by COVID-19 or otherwise, family caregiver access]

1. During COVID, what are the most pressing needs that exist in long-term care homes related to advance care planning?
2. What impact do you feel having an Advance Care Plan in place while in Long Term Care would have on the quality of care a resident would receive?
3. What, if any, specific considerations should we be aware of when considering advance care planning within your region?

**Implementation of Goals of Care**

1. How has COVID-19 impacted the implementation of goals of care in long-term care?

[If needed: definition of goals of care: is a medical order used to describe and communicate the general aim or focus of care including the preferred location of that care.]

[*Probes*: Impact of: staffing levels, staff working at different homes, organizations beyond a single LTC home, linkages with hospital or community-based response teams, infection prevention and control measures, meeting the basic care needs of residents, including feeding and bathing, medical assessment and treatment of residents affected by COVID-19 or otherwise, family caregiver access]

1. During COVID, what are the most pressing needs that exist in long-term care homes related to goals of care?
2. What, if any, specific considerations should we be aware of when considering goals of care within your region?

**Tools and Resources**

1. What tools and resources do you currently use to help implement:
   1. a palliative approach to care
   2. Advance Care Planning
   3. Goals of care
2. Would you be willing to share these tools and resources with us to add them into a LTC specific repository of information for HCPs?
3. [If yes]: Can you please email them to me?
4. Outside of your own organization, are there any other tools that you are aware of that would be useful for us to consider including in a LTC specific repository of information for HCPs?
   1. [If yes] can you please describe the tools to me?
   2. Do you have a copy that you could share? OR the contact information for the organization that I can request the document from?
5. What new tools and/or resources are needed now given the demands of COVID to help you implement:
6. a palliative approach to care
7. ACP
8. goals of care
9. Do you have any suggestions on how these tools and resources could be created for:
10. Family members?
11. Residents?
12. Health Care Providers and other workers within the Long-Term Care environment?
13. What would be the best way to disseminate tools and resources? [probe: to reach residents, to reach families, to reach health care providers]
14. Where could they be placed?
15. How can we get the message out that they are available?
16. Do you have anything else that you would like to share about your experience implementing a palliative approach to care, including advance care planning and goals of care, in the environment of COVID-19.
